# Pilot, randomized, placebo-controlled clinical field study to evaluate the effectiveness of bupivacaine liposome injectable suspension for the provision of post-surgical analgesia in dogs undergoing stifle surgery

**DOI:** 10.1186/s12917-016-0798-1

**Published:** 2016-08-17

**Authors:** B. Duncan X. Lascelles, Lesley C. Rausch-Derra, Jessica A. Wofford, Margie Huebner

**Affiliations:** 1 Department of Clinical Sciences, Comparative Pain Research Laboratory, College of Veterinary Medicine, North Carolina State University, Raleigh, NC USA; 2 Department of Clinical Sciences, Comparative Medicine Institute, College of Veterinary Medicine, North Carolina State University, Raleigh, NC 27606 USA; 3 Center for Pain Research and Innovation, Dental School, UNC Chapel Hill, Chapel Hill, NC USA; 4 Department of Anesthesiiology, Center for Translational Pain Medicine, Duke University, Durham, NC USA; 5 Aratana Therapeutics, Inc., Leawood, KS 66211 USA; 6 ClinData Services, Inc., 6716 Holyoke Court, Fort Collins, CO 80525 USA

**Keywords:** Bupivacaine liposome injectable suspension, Dog, Pain, Cruciate surgery

## Abstract

**Background:**

Local anesthetics are an important component of perioperative pain management, but the duration of action of available products is limited. We hypothesized that a single local infiltration of a novel bupivacaine liposome injectable suspension (AT-003) would provide clinically effective analgesia over a 72-h period.

In a masked, randomized, placebo-controlled, multi-center pilot field study, dogs undergoing lateral retinacular suture placement for cranial cruciate insufficiency were randomly assigned to surgical site infiltration with AT-003 (5.3 mg/kg) or an equivalent volume of saline. Infiltration of the surgical site was done prior to closure. Primary outcome measure was the Glasgow Composite Measure Pain Scale (CMPS-SF) assessed prior to surgery and at 2, 4, 8, 12, 24, 30, 36, 48, 54, 60 and 72 h following surgery by trained individuals. Provision for rescue analgesia was employed. Repeated measures analysis of variance were utilized to test for possible differences between treatment groups and a success/failure analysis was also employed, based on the need for rescue analgesia.

**Results:**

Forty-six dogs were enrolled and evaluated. For CMPS-SF scores there was a significant overall treatment effect (*p* = 0.0027) in favor of AT-003. There were significantly more successes in the AT-003 group compared to placebo over each time period (*p* = 0.0001 for 0–24 h, *p* = 0.0349 for 0–48 h, and *p* = 0.0240 for 0-72 h). No significant adverse events were seen.

**Conclusions:**

AT-003 (bupivacaine liposome injectable suspension) provided measurable local analgesia over a 72-h period following post-stifle surgery surgical site tissue infiltration. Further work is indicated to develop this product for clinical use.

**Electronic supplementary material:**

The online version of this article (doi:10.1186/s12917-016-0798-1) contains supplementary material, which is available to authorized users.

## Background

Perioperative analgesia has become recognized as an important ethical responsibility of veterinarians over the last three decades. Clinical evidence in dogs indicates that multimodal analgesia provides the most effective relief from postoperative pain [1]. One of the most effective means of preventing the transduction and transmission of nociceptive signals is through the use of local anesthetics. Indeed, it is the authors’ opinion that the only currently available analgesics that can completely block perioperative pain are the local anesthetics. Despite their potential efficacy, the relief provided by currently available local anesthetics is of limited duration, and this may be one factor contributing to the currently relatively low use of local anesthetics in small animal practice [2]. Bupivacaine has the longest reported activity, potentially providing analgesia for as long as 6–7 h [3–5]. However, currently the only method in veterinary medicine to extend the action of bupivacaine beyond this involves using a wound catheter, and instilling bupivacaine approximately every 6 h into the wound [6, 7].

In 2011 the FDA approved an extended-release formulation of bupivacaine, DepoFoam® bupivacaine^1^ for use as a single-dose infiltration into the surgical site to effect postsurgical analgesia in human surgical patients. The DepoFoam technology used in this product consists of multivesicular liposomes encapsulating aqueous bupivacaine. The liposomes are microscopic structures made of nonconcentric lipid bilayers designed such that bupivacaine is gradually released from vesicles over 96 h as the lipid bilayers break down. The lipids making up the bilayer structures consist of phospholipids, cholesterol and triglycerides, and importantly do not contain lecithin which has been associated with tissue necrosis and toxicity [8]. Bupivacaine liposome injectable suspension (Depofoam bupivacaine) has been extensively studied in dogs as part of the development for human use [9, 10]. Bupivacaine liposome injectable suspension, known as AT-003^2^ is currently being investigated for use in veterinary patients. In a preliminary laboratory study to assess the analgesic properties of AT-003 following tissue infiltration around the site of stifle arthrotomy in beagle dogs, a dose of 5.3 mg/kg was determined to provide adequate analgesia for at least 24 h post-surgery (unpublished data). The current report describes a pilot field study evaluating the post-operative analgesia provided by AT-003 at a dose of up to 5.3 mg/kg administered by tissue infiltration just prior to closure following cranial cruciate ligament (CCL) surgery in client-owned dogs, using subjective, in-clinic assessments of pain.

We hypothesized that 5.3 mg/kg of bupivacaine liposome injectable suspension would provide clinically effective analgesia, as measured using subjective clinical assessment, over a 72-h period following stifle surgery in dogs, when compared to a saline placebo.

## Results

**Fig. 1:**
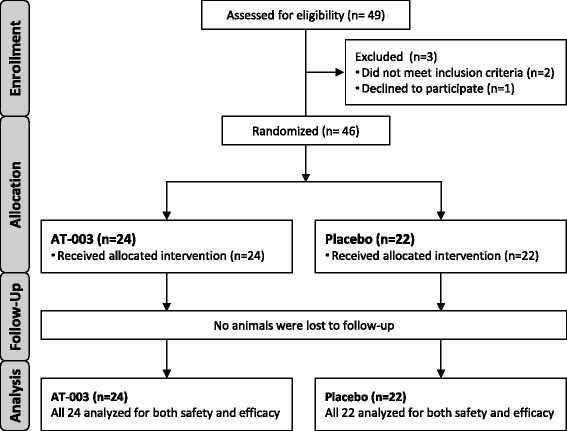
Study participant flow diagram

Three (3) investigative sites screened and enrolled cases. There were forty-nine (49) dogs screened and forty-six (46) dogs enrolled in the study over the period April 2014 to September 2014 and the study concluded when the target number of dogs was enrolled. All forty-six (46) dogs enrolled in the study were included in both the efficacy and safety evaluation (see flow diagram, Fig. 1). Twenty-four (24) dogs were treated with AT-003 and twenty-two (22) dogs with saline. The mean (range) age was 7.47 years (2.1–13.3) for the AT-003 group and 6.71 years (1.0–10.4) for the saline placebo group. Group characteristics are summarized in Table 1.

**Table 1:** Per protocol population demographics of enrolled dogs in the AT-003 and saline placebo groups

Characteristic		AT-003 (*n* = 24)	Saline placebo (*n* = 22)
Age (years)	Mean	7.47	6.71
	Min, Max	2.08, 13.33	1.00, 10.42
Weight (kg)	Mean	26.54	22.57
	Min, Max	3.2, 79.3	2.4, 44.8
Sex	Female	0	0
	Female spayed	13	11
	Male	1	0
	Male castrated	10	11

### CMPS-SF scores

The mean total CMPS-SF scores (+/− SEM) at the baseline (pre-surgery) evaluation were 1.58 (0.22) and 1.18 (0.20) for AT-003 and saline placebo, respectively. Following surgery, total CMPS-SF scores (Table 2) were consistently lower at all post-operative time points (except for 72 h) for the AT-003 treated dogs compared to saline placebo treated dogs, with a significant overall treatment effect (*p* = 0.0027) in favor of AT-003. At 72 h, the scores were similar, but only two dogs remained in the saline placebo group (note: at 60 h data were missing from one placebo dog). There was no treatment by time effect (*p* = 0.8041), however there were only a small number of subjects remaining in the saline placebo group after 12 h. When site effect and treatment by site interaction were entered into the model, the overall treatment effect was again statistically significant (*p* = 0.0036), with lower CMPS-SF scores in the AT-003 group.

**Table 2:** Total mean (SEM) CMPS-SF scores by treatment group and time point

Time point (hours)	AT-003		Saline placebo	
	Number of dogs evaluated	Mean (+/SEM) CMPS-SF	Number of dogs evaluated	Mean (+/−SEM) CMPS-SF
0	24	1.58 (0.22)	22	1.18 (0.20)
2	24	4.38 (0.47)	22	6.82 (0.72)
4	22	3.36 (0.35)	16	6.56 (0.82)
8	21	4.24 (0.43)	9	7.00 (1.11)
12	19	4.21 (0.66)	5	6.20 (1.93)
24	14	3.50 (0.69)	4	4.50 (1.32)
30	11	2.64 (0.43)	3	5.67 (1.20)
36	10	2.20 (0.29)	3	3.33 (1.20)
48	10	2.30 (0.68)	3	4.67 (2.19)
54	9	2.44 (0.53)	2	3.00 (2.00)
60	9	2.00 (0.53)	1	5.00 (NA)
72	9	2.11 (0.48)	2	2.00 (0.00)

*NA* Not Applicable

### Surgical site manipulation scores

The mean Surgical Site Manipulation Scores (SSMS) (+/−SEM) at the baseline (pre-surgery) evaluation were 0.42 (0.1) and 0.68 (0.21) for AT-003 and saline placebo, respectively. Following treatment, the mean scores for AT-003 were less than those for placebo at each evaluation time point except at 12, 54, 60 and 72 h (Table 3), however, there were only two placebo dogs left in the study at 54 h. The overall treatment effect showed no statistically significant difference between treatment groups (*p* = 0.0941), however when site effect and treatment by site interaction were entered into the model, the overall treatment effect was statistically significant (*p* = 0.0312), with lower surgical site manipulation scores in the AT-003 group.

**Table 3:** Total Mean (SEM) Surgical Site Manipulation Scores by Treatment Group & Time Point

Time point (hours)	AT-003		Saline Placebo	
	Number of dogs evaluated	Mean surgical site manipulation (SEM)	Number of dogs evaluated	Mean surgical site manipulation (SEM)
0	24	0.42 (0.10)	22	0.68 (0.21)
2	24	1.13 (0.31)	22	2.32 (0.30)
4	22	0.95 (0.27)	16	2.56 (0.32)
8	21	1.33 (0.30)	9	2.11 (0.45)
12	19	1.47 (0.30)	5	1.40 (0.51)
24	14	1.43 (0.39)	4	1.50 (0.50)
30	11	0.82 (0.33)	3	1.67 (0.67)
36	10	0.70 (0.30)	3	1.00 (1.00)
48	10	0.90 (0.35)	3	1.00 (1.00)
54	9	0.67 (0.37)	2	0.00 (0.00)
60	9	0.33 (0.24)	1	0.00 (NA)
72	9	0.67 (0.29)	2	0.00 (0.00)

*SEM* standard error of the mean, *NA* not applicable

### Success/Failure analysis

Dogs receiving AT-003 were more likely to remain in the study (that is, not be rescued due to insufficient pain relief) following the surgical procedure than dogs receiving the saline placebo. At 72 h post-operatively, 9 of 24 AT-003 dogs and 2 of 22 placebo dogs remained in the study. Overall treatment success at each time period was statistically significantly different between the groups (*p* = 0.0001 for 0-24 h; *p* = 0.0349 for 0–48 h; and *p* = 0.0240 for 0–72 h) with more dogs being designated treatment successes in the AT-003 treatment group compared to the saline placebo group in each time period (Table 4). The odds of success were almost 13 times greater in the AT-003 group compared to the placebo group in the first 24 h; 4.5 times greater over the 0–48 h period, and 6 times greater over the 0–72 h period.

**Table 4:** Summary results of the success/failure analysis. Dogs deemed failures in one time period were carried forward into the next time period, where they were also categorized as failures. The Odds of success (and 95 % Confidence Interval, CI) are tabulated

Time interval	AT-003		Saline placebo	Chi-square *p*-value	Odds ratio
0–24 h	Success	19 (79.2 %)	5 (22.7 %)	0.0001	12.92 (3.18, 52.48)
	Failure	5 (20.8 %)	17 (77.3 %)		
0–48 h	Success	10 (41.7 %)	3 (13.6 %)	0.0349	4.52 (1.05, 19.54)
	Failure	14 (58.3 %)	19 (86.4 %)		
0–72 h	Success	9 (37.5 %)	2 (9.1 %)	0.0240	6.0 (1.13, 31.94)
	Failure	15 (62.5 %)	20 (90.9 %)		

### Adverse events

AT-003 appeared to be well tolerated. Adverse events were reported in 5 dogs during the study, three in the AT-003 group and two in the saline placebo group, all from a single site. All were classified with an “unlikely” or “unknown” relationship to study treatment. One dog in the AT-003 treatment group had mild bradycardia (heart rate was 56 beats per minute, as compared to 96 beats per minute at the pre-enrollment physical examination) detected 4 hours after surgery (at time of rescue). Another dog in the AT-003 treatment group vomited on a single occasion, and this resolved with no treatment. A third dog in the AT-003 group was found to have a small excoriated patch on its nose, considered unlikely to be treatment related, and rather resulting from trying to exit the cage. In the saline placebo group, a flea was found on one dog, and another had an episode of diarrhea with fresh blood, which resolved without treatment.

## Discussion

The study hypothesis was supported, with the results indicating measurable analgesic effects of AT-003 (bupivacaine liposome injectable suspension) compared to placebo, over a 72-h period following surgical site tissue infiltration in 46 dogs undergoing cranial cruciate ligament (CCL) surgery. The odds of success (in terms of not needing rescue) were dramatically greater in the AT-003 group than the placebo group. It is possible that some dogs may have been inappropriately designated as comfortable when in fact pain was present, potentially due to the carry-over effects of the acepromazine sedation in the early postoperative period, or due to a lack of sensitivity of the pain assessment tool. However, in these respects, both groups should have been equally affected, making the comparison valid.

Pain is difficult to measure, and although the assessment tool used in the present study has been widely used, it has never undergone criterion validation. However, one measure of the success in measuring pain in placebo-controlled studies is the rescue rate in the placebo group. In the current study, it was 77 % within 24 h, and 91 % over the 72 h of the study, suggesting inadequate pain relief was being detected, and giving one further confidence that AT-003 provided clinically measurable pain relief. If one assumes surgery is associated with pain, then the lack of rescue of 2/22 dogs in the placebo group would be considered a failure of the assessment system. However, the approach to assessment and rescue in our study appears to have been comparatively successful when looked at other published studies. In a study of dogs undergoing cruciate surgery [11], only 11 % (1 of 9 dogs) was rescued in the 24 h following surgery. In a study of dogs undergoing soft tissue surgery [12], only 9 of 16 dogs were rescued over a 72-h period postoperatively. The lack of intra-operative analgesia provision (beyond preoperative hydromorphone, which all dogs received) was very deliberate due to how difficult pain is to measure. Given how insensitive our current assessment tools are, the more background treatments that are administered, the more difficult it becomes to assess whether a novel analgesic provides any benefit. Future work, hopefully with more sensitive, and ideally objective, outcome assessments, could evaluate the effect size of this product versus other treatments.

There are few data as to how long post-surgical pain persists in animals, and this time period will vary with the type of surgical procedure performed. The recommended time period for administration of analgesics post-operatively varies from days to weeks, depending on the surgery performed, but expert opinion emphasizes the first 72 h following surgery as a critical time period when analgesics should be administered [13]. Post-surgical pain can generally be well controlled while an animal is hospitalized using injectable opioids, ketamine, cyclooxygenase inhibiting NSAIDs and local anesthetics. However, with most veterinary patients being discharged from the veterinary hospital the same day, or within 24 h following surgery, there is a need to bridge between analgesics provided in the hospital and effective pain relief in the home environment. Currently in the United States, only oral cyclooxygenase inhibiting NSAIDs and transdermal fentanyl liquid^3^ have been approved for post-surgical analgesia in dogs. Beyond these options, there are unapproved fentanyl patches manufactured for human use, which have been suggested to be efficacious [14] and tramadol is widely used, despite little evidence of efficacy [15–18].

Overall, there is a clear unmet need for effective analgesic products that can be administered in the clinic, and provide pain relief for the crucial first few days following surgery in the home environment. This pilot study suggests that bupivacaine liposome injectable suspension may directly address this unmet need, providing up to 72 h of analgesia. Though statistically greater than placebo, not all of the AT-003 dogs were successes out to 72 h. In this study, surgeons were not instructed to infiltrate around the anchor points of the suture material. In future studies, infiltration into all the tissue layers as well as around the anchor points for suture or around implants will be performed. Bupivacaine liposome injectable suspension will need to be administered as part of a multimodal approach as the anesthetic wears off, and indeed, this is consistent with the current recommendations for the treatment of postoperative pain [19, 20]. The prolonged duration of continuous action may also prevent episodes of breakthrough pain.

The current study was a pilot study, performed in dogs undergoing a single type of surgery, lateral retinacular suture placement for cruciate insufficiency. As such, the generalizability of these results to the larger population of dogs undergoing a variety of orthopedic surgeries and other surgical procedures is unknown. However, given the demonstrated efficacy in human clinical studies in both orthopedic [21] and soft tissue [22] surgery, it is highly likely that AT-003 will prove efficacious in dogs undergoing a wide variety of surgical procedures.

The current study employed direct injection of bupivacaine liposome injectable suspension into the surgical wound, and this may raise concerns about wound healing and wound infection. While there is some evidence that local anesthetics alter the cellular events of early tissue healing, there does not appear to be a clinically significant impact on wound healing or mechanical wound strength in preclinical animal studies [23] or humans. Infection rates following continuous local anesthetic installation into wounds found that reported wound infection rates were similar between active (0.7 %) and control groups (1.2 %) in humans undergoing surgery [24]. In the present study, no wound related adverse effects were seen, which mirrors the extensive pre-clinical work in dogs that has been performed with this product. In a dog model of inguinal hernia repair, bupivacaine liposome injectable suspension was infiltrated into the surgical site at doses ranging from 9 to 25 mg/kg and a mild granulomatous inflammatory response was seen histologically, that had not resolved by 2 weeks following infiltration, but was not considered indicative of any adverse effect on wound healing and the wound healing findings were similar to control (saline or bupivacaine HCL) treated animals [9]. In the present study, one dog was found to have bradycardia 4 h following administration of AT-003. In previous work, repeated doses of bupivacaine liposome injectable suspension were administered to dogs and the dogs evaluated for cardiotoxicity [25]. Doses of 9, 18 and 30 mg/kg bupivacaine liposome injectable suspension, 9 mg/kg bupivacaine HCl, or a volume-equivalent dose of saline, were administered into the subcutaneous tissue over the scapulae twice weekly for 4 weeks. No clinical signs consistent with central nervous system toxicity were seen and no electrocardiogram abnormalities were detected [25].

Dilution in this study was allowed based on the human experience and volumes used in previous canine knee surgery studies. Dilution does not impact efficacy in humans [26], and that appeared to be the case in this study as well. Most surgeons administered the drug in the undiluted state. The one surgeon who did dilute did so such that the total volume was divisible by four, facilitating a 25, 50 and 25 % tissue infiltration distribution.

## Conclusions

Further work is indicated to develop this product for clinical use. This product has significant potential to address an important clinical need - the provision of extended duration local wound analgesia.

## Methods

### Design

The study was a masked, randomized, placebo-controlled, multi-center pilot field study, conducted under Good Clinical Practice Guidelines. The study was performed as part of the development program of AT-003 for FDA approval in dogs. The study was conducted according to the protocol and in compliance with Good Clinical Practice (VICH) Guideline GL9, International Cooperation on Harmonization of Technical Requirements for Registration of Veterinary Medicinal Products (VICH), June 2000, and the FDA Center for Veterinary Medicine (CVM) Guidance for Industry #85, May 2001. The study protocol was approved by Aratana’s internal review board. Each owner gave written informed consent prior to dogs being enrolled into the study, and best practices for veterinary care and patient management were followed throughout the study.

### Study population

Owned pet dogs of any breed or gender, greater than 5 months of age, deemed to require surgery for cruciate ligament insufficiency, were recruited to the study.

### Inclusion criteria

Dogs were required to have been diagnosed with cranial cruciate ligament insufficiency within the previous 4 months, and not to have received systemic anti-inflammatories within 7 days of the surgical procedure. Dogs of a fractious demeanor, or who had an uncontrolled endocrine or systemic disorder, or who had had surgery within the previous 14 days were not eligible.

### Study protocol overview

**Fig. 2:**
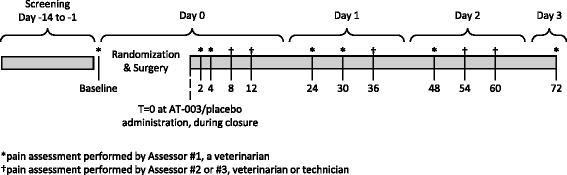
Study Protocol Outline

The study evaluated the post-operative analgesia provided by AT-003 at a dose of 5.3 mg/kg administered by tissue infiltration just prior to closure following unilateral cranial cruciate ligament (CCL) surgery in dogs. The study protocol is outlined in Fig. 2. Dogs were randomly assigned to one of the two treatment groups and AT-003 (5.3 mg/kg) or saline placebo (1.0 mL/kg) was administered into the wound bed at the end of surgery, prior to and during closure of the wound. Both AT-003 and saline were injected directly into tissues, using a moving needle technique.

All dogs underwent the lateral retinacular suture procedure, including stifle arthrotomy. The primary outcome measure was the Glasgow Composite Measure Pain Scale (CMPS-SF). Assessments were performed prior to premedication/surgery following a 2-h acclimation period, and at 2, 4, 8, 12, 24, 30, 36, 48, 54, 60 and 72 h following surgery by veterinarians and veterinary technicians trained on the use of the CMPS-SF. After the CMPS-SF assessment was completed, a Surgical Site Manipulation Score (secondary outcome measure) was assessed by standardized manipulation of the stifle joint that had undergone surgery (Table 5). Dogs were administered rescue analgesia (choice of rescue was at the discretion of the veterinarian) if the CMPS-SF score was greater than 8 or if the assessors felt additional analgesia was needed. Following either of these, dogs were removed from the study. Everyone involved in the assessments was trained in the use of the assessment tools through explanation and discussion of the tools during a single ‘classroom’ style training session run by the Contract Research Organization, and the document describing the definitions of expressions used in the CMPS-SF. All assessors had prior experience of using the CMPS-SF in clinical studies. No more than 3 assessors were permitted to do the assessments on any one dog. Different sites were not calibrated on the use of the CMPS-SF, and nor were any measures put in place to confirm consistency of scoring.

**Table 5:** Surgical Site Manipulation scoring system. At each relevant assessment time point, the joint was gently palpated around the surgical incision, and then the joint was put through a passive range of motion, flexing and extending the joint

Manipulate the joint through a normal range of motion, and score the patient as per below:	
Score	Response
0	Does not notice manipulation.
1	Orients to site on manipulation, does not resist.
2	Orients to site, may lick, slight objection to manipulation
3	Withdraws from manipulation, may vocalize, excessive licking
4	Tries to escape from manipulation, or prevent manipulation, may bite or show aggression.

Safety assessments (secondary outcome measures) were made using clinical observations and behavioral observations at all assessment time points, and physical examination findings at 72 h or following rescue. No specific guidance on what to look for in the clinical observations was given; the physical examination was a standard physical examination covering evaluation of all body systems. Owners were also called within 4–7 days following surgery to capture any adverse events that occurred following discharge.

### Anesthetic protocol

Acepromazine (0.05 mg/kg) was administered intravenously (IV) or intramuscularly (IM) with a maximum total dose of 3.0 mg regardless of body weight. The doses of acepromazine used in the study were based on our experience with the drug. At least 15 min after acepromazine administration, hydromorphone (0.1 mg/kg) was administered IV. Anesthesia was induced with propofol, and maintained on isoflurane carried in oxygen, delivered via an endotracheal tube and a rebreathing circuit. Additional local/regional anesthesia (epidural, nerve block, intra-articular etc.) was not permitted, nor were any other analgesics. Fluids were administered intravenously during anesthesia.

### Randomization to treatment groups

Dogs were randomly assigned to one of the two treatment groups and treated post-CCL surgery with AT-003 (5.3 mg/kg, equivalent to 0.4 mL/kg) or saline (1.0 mL/kg). Surgeons were allowed to dilute AT-003 up to 15 times, if needed, in order to cover the surgical area, with a recommended volume of 0.4–1.0 mL/kg based on previous studies. Randomization was performed using the Prelude Dynamics VISION™ electronic data capture (EDC) system. The VISION randomization framework generated each random treatment from a pre-computed table of treatments for each site. The randomization calculation maintained equal size groups per site and was calculated based on the current patient population. Subjects were randomized in blocks of two, stratified by site.

### Masking procedures

One individual at each site was designated the Dispenser, and they documented the assignment of dogs to treatment group and kept the related documentation in a secure site, away from all other personnel. The Dispenser and the veterinarian performing the surgery had knowledge of which treatment group each dog was assigned. Blinded personnel were not given access to the treatment or randomization forms. The veterinarian or trained technician performing the assessments, sponsor, statistician and any other personnel involved in clinical evaluations and observations remained blinded to treatment during the study and until data base lock. The Owners were also blinded to treatment.

### Tissue infiltration procedure

AT-003 or saline was injected slowly into the tissues using a 1–1.5 in. long needle, using a ‘moving needle technique’, in which the needle is inserted to near the hub and the material is injected as the needle is pulled out. The needle is inserted at varying angles depending on how the tissue is best approached in order to deposit the injectate within the tissues. In general, this means inserting the needle approximately parallel to the surface of the skin. The injections are repeated to create an area of infiltrated tissue around the whole wound, and at all levels of the wound. Approximately 25, 50 and 25 % of the AT-003 or saline was injected into the tissues around the joint capsule, the fascial tissue, and the sub-cuticular tissue, respectfully. The injections were performed such that the whole wound, at all levels, was infiltrated with AT-003 or saline. Dilution of AT-003 with normal saline was permitted and was utilized for 12 of the AT-003 treated dogs, all at one site, with a maximal dilution of 1:1 AT-003 to saline.

### Outcome measures

The primary outcome measure was the Glasgow Composite Measure Pain Scale (Short Form) (CMPS-SF), and secondary outcome measures were a Surgical Site Manipulation Score and an evaluation of adverse events.

### CMPS-SF

The Glasgow Composite Measure Pain Scale-Short Form (CMPS-SF), (http://www.newmetrica.com/acute-pain-measurement/download-short-form-painquestionnaire-for-dogs/) is a subjective clinical metrology instrument used by animal caretakers to evaluate perioperative pain in a practice setting that was developed at the University of Glasgow, UK [27, 28]. The expressions used in the CMPS-SF were defined in order to assist in training of sites, and are detailed in Additional file 1. Data were captured electronically, and thus the electronic version of the CMPS-SF differed from the published paper version. The layout of the electronic version is illustrated in Additional file 2: Figure S1.

### Surgical site manipulation score (SSMS)

A simple descriptive scale, scored on a 0–4 scale (Table 5) was used for the assessment of surgical site pain associated with cruciate surgery. The scale was constructed by one of the authors, and is similar to wound palpation scoring systems used previously [7].

### Adverse events

An adverse event was defined as any observation, undesirable experience, reaction or side effect in animals that was unfavorable and unintended and occurred after the use of AT-003 or placebo, whether or not considered to be treatment related. A serious adverse event was defined as any adverse event that resulted in death, was life-threatening, or resulted in persistent or significant disability/incapacity, although no serious adverse event occurred during this study.

### Rescue analgesia

Dogs were removed from the study and administered rescue analgesia if the CMPS-SF score at a designated assessment time point was greater than 8 or at any time (assessment time point or at any time in between) if the assessing veterinarian felt additional analgesia was needed. The intervention score of 8 was based on one investigator’s (BDXL) experience with the CMPS-SF, and a lack of data to substantiate the suggestion of an intervention score of 6 (http://www.newmetrica.com/acute-pain-measurement/download-short-form-painquestionnaire-for-dogs/). No dog in the study was removed for a reason other than rescue analgesia.

### Data capture

Most data were captured electronically using an electronic data capture (EDC) system VISION developed and supported by Prelude Dynamics, LLC.

### Power analysis and statistical analysis

The enrollment target was at least 40 evaluable cases (20 AT-003 and 20 placebo) per the study randomization schedule across all sites. An evaluable sample size of 20 AT-003 and 20 placebo cases was determined to be able to provide more than 90 % power (alpha = 0.05, 2-sided) to detect a difference between treatment groups of 0.37 (pooled standard deviation = 0.30) in the CMPS-SF assessment at 24 h post-administration. These calculations were based on results from a preliminary laboratory study to assess the analgesic properties of AT-003 following tissue infiltration around the site of stifle arthrotomy in beagle dogs.

To be eligible for the statistical analysis, each site must have enrolled and completed a minimum of 2 evaluable cases (at least 1 of each treatment). For total CMPS-SF and Surgical site manipulation scores, repeated measures analysis of variance were utilized to test for possible differences between treatment groups. SAS/STAT Proc MIXED were implemented with the model containing terms for treatment group, time point and the interaction of treatment by time point. The baseline value was included in the model as a covariate. If the treatment group by time point interaction was found to be statistically significant, then treatment effects were evaluated for each time point. The covariance structure that provided the smallest Akaike’s Information Criterion value was used. Covariance structures that were tested include Compound Symmetry (CS), heterogeneous CS (CSH), first-order autoregressive [AR(1)] and heterogeneous autoregressive [ARH(1)]. Additionally, exploratory analysis used a repeated measures analysis of variance that included site effect and treatment by site interaction in the model.

A success/failure analysis was also employed, with success defined as no pain intervention over the intervals of 0–24 h, 0–48 h and 0–72 h. The number and percentage of dogs that were a success were presented for each time interval by treatment group. The chi-square test was utilized to test for possible differences between treatment groups, and odds ratios described.

## Additional files

Additional file 1: Definitions of the expressions used in the CMPS-SF. In order to facilitate consistency in the way the CMPS-SF was used, the expressions used in the CMPS-SF were defined, and this file contains those definitions. (DOCX 102 kb) Additional file 2: Figure S1. Layout of the electronic version CMPS-SF. The electronic version CMPS-SF differed from the published paper version, and the layout of the electronic version is shown in this Additional file. (TIFF 5625 kb)

## Abbreviations

CCL: Cranial cruciate ligament
CMPS-SF: Glasgow composite measure pain scale (short form)
EDC: Electronic data capture
IV: Intra-venous
SSMS: Surgical site manipulation scores

## Footnotes

1 Exparel, Pacira Pharmaceuticals, Inc., Parsippany, NJ.

2 Nocita, Aratana Therapeutics, Inc., Leawood, KS.

3 Recuvyra™, Elanco.
