# Supplementary material for: Pilot, randomized, placebo-controlled clinical field study to evaluate the effectiveness of bupivacaine liposome injectable suspension for the provision of post-surgical analgesia in dogs undergoing stifle surgery
Source: BMC Vet Res. 2016 Aug 17;12:168. doi: 10.1186/s12917-016-0798-1 (PMC4988028; doi:10.1186/s12917-016-0798-1)
Supplement: Additional file 1: — Definitions of the expressions used in the CMPS-SF. In order to facilitate consistency in the way the CMPS-SF was used, the expressions used in the CMPS-SF were defined, and this file contains those definitions. (DOCX 102 kb) [file 12917_2016_798_MOESM1_ESM.docx]

### Appendix X.

### Definitions of Expressions Used in the CMPS-SF

The following definitions were used to differentiate behaviors within each CMPS-SF (<http://www.newmetrica.com/cmps/eng/download/CMPS_Eng.pdf>) behavior category:

Vocalization (i):

- Quiet: no vocalizing
- Whimpering: often quiet, short, high pitched sound, frequently closed mouth (whining)
- Crying: extension of the whimpering noise, louder and with open mouth
- Groaning: low moaning or grunting deep sound, intermittent
- Screaming: animal making a continual high pitched noise, inconsolable, mouth wide open

Attention to wound or painful area (surgical site) (ii):

- Ignoring: paying no attention to wound or painful area (surgical site)
- Looking: turning head in direction of wound or painful area (surgical site)
- Licking: using tongue to stroke area of wound or painful area (surgical site)
- Rubbing: using paw or kennel floor, etc. to stroke wound or painful area (surgical site)
- Chewing: using mouth and teeth on wound or painful area (surgical site), pulling stitches

Mobility (iii):

- Normal: gets up and walks with no alteration from normal walk
- Lame: irregular gait, uneven weight bearing when walking
- Slow: slow to get up or walk but not stilted in movement
- Reluctant: needs encouragement to get up or walk
- Stiff: stilted gait, slow to rise or walk, may be reluctant to move

Response to touch (iv):

- Does nothing: accepts firm pressure near wound or painful area (surgical site) with no reaction
- Flinch: wound or painful area (surgical site) is quickly moved away from stimulus either before or in response to touch
- Growl: emits a low prolonged warning sound before or in response to touch
- Guard: pulls wound or painful area (surgical site) away from stimulus, tenses local muscles in order to protect from stimulus
- Snap: tries to bite observer before or in response to touch
- Cry: a short vocal response; looks at area and opens mouth, emits a brief sound

Demeanor (v):

- Content: interested in surroundings, positive interaction with observer, responsive and alert
- Bouncy: tail wagging, jumping in kennel often vocalizing with a happy and excited noise
- Quiet: sitting or lying still, no noise, will look when spoken to, but not respond
- Indifferent: not responsive to surroundings or observer
- Nervous: eyes in continual movement, often head and body movement, jumpy
- Anxious: worried expression, eyes wide with white showing, wrinkled forehead
- Fearful: cowering away, guarding body and head
- Depressed: dull demeanor, not responsive, shows reluctance to interact
- Non-responsive to stimulation: cannot be stimulated to wag tail or interact with observer

Posture / Activity (vi):

- Comfortable: animal resting and relaxed, no avoidance or abnormal body position evident, settled, remains in same body position, at ease
- Unsettled: animal resting but with avoidance or abnormal body position.
- Restless: moving body position, circling, pacing, shifting body parts
- Hunched: when animal is standing, its back forms a convex shape with abdomen tucked up, or, back in a concave shape with shoulders and front legs lower than hips
- Tense: animal appears frightened or reluctant to move jaw, overall impression of tight muscles; animal can be in any body position
- Rigid: animal lying in lateral recumbency, legs extended or partially extended in a fixed position
