# Supplementary figures and images for: Pilot, randomized, placebo-controlled clinical field study to evaluate the effectiveness of bupivacaine liposome injectable suspension for the provision of post-surgical analgesia in dogs undergoing stifle surgery
Source: BMC Vet Res. 2016 Aug 17;12:168. doi: 10.1186/s12917-016-0798-1 (PMC4988028; doi:10.1186/s12917-016-0798-1)

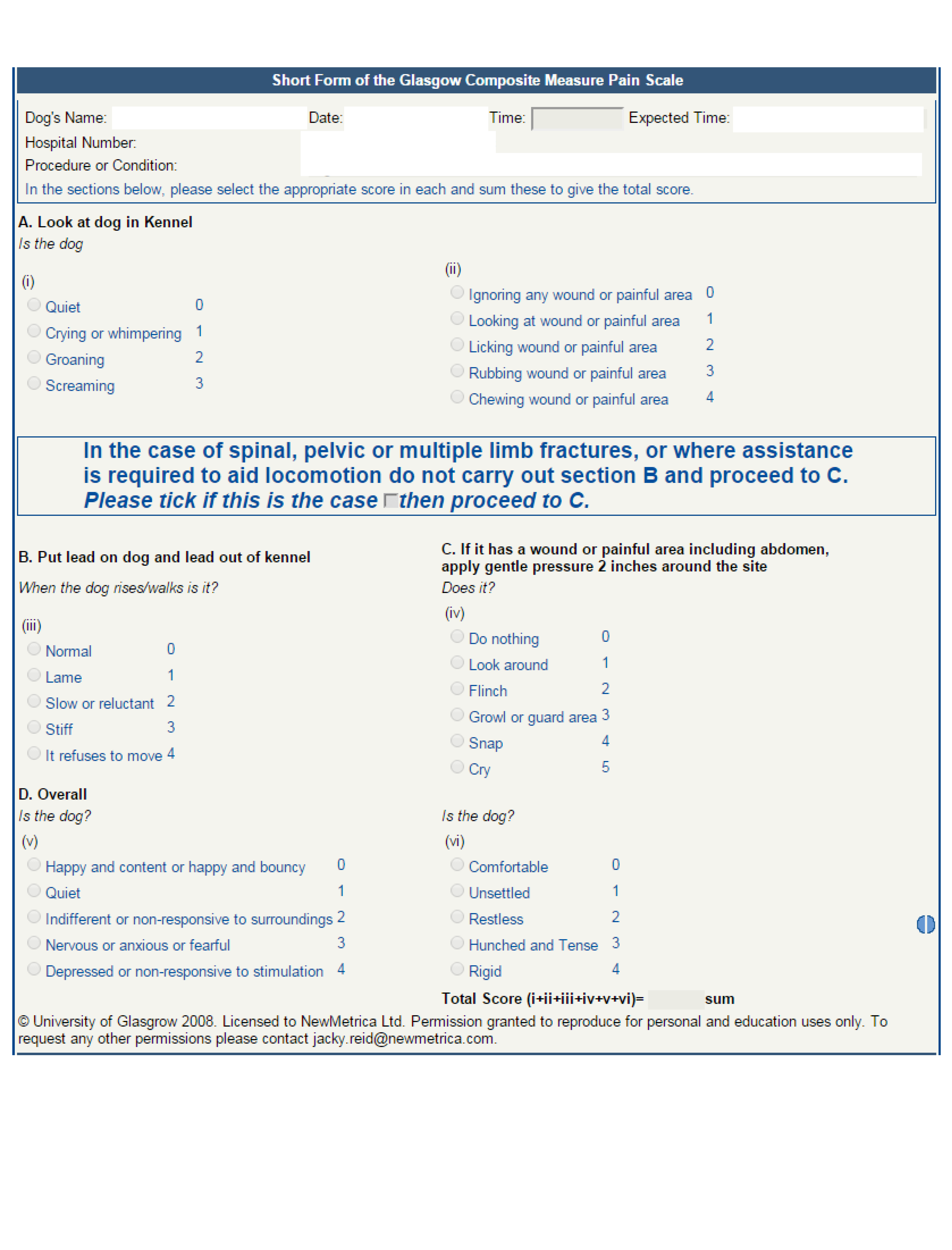

Supplement: Additional file 2: Figure S1. — Layout of the electronic version CMPS-SF. The electronic version CMPS-SF differed from the published paper version, and the layout of the electronic version is shown in this Additional file. (TIFF 5625 kb) [file 12917_2016_798_MOESM2_ESM.tiff]
